# Supplementary material for: CRISPRi-Linked Multi-Module Negative Feedback Loops to Address Winner-Take-All Resource Competition
Source: bioRxiv. 2025 May 16:2025.05.15.654351. Preprint. [Version 1] doi: 10.1101/2025.05.15.654351 (PMC12132280; doi:10.1101/2025.05.15.654351)
Supplement: Supplement 1 [file media-1.pdf]

Supplementary Information

CRISPRi-Linked Multi-Module Negative Feedback Loops to Address Winner-Take-All Resource Competition

Sadikshya Rijal, Kylie Standage-Beier, Rong Zhang, Austin Stone, Abdelrahman Youssef, Xiao Wang, Xiao-Jun Tian*

[1] School of Biological and Health Systems Engineering, Arizona State University, Tempe, Arizona, 85281, United States.

*Correspondence: xiaojun.tian@asu.edu

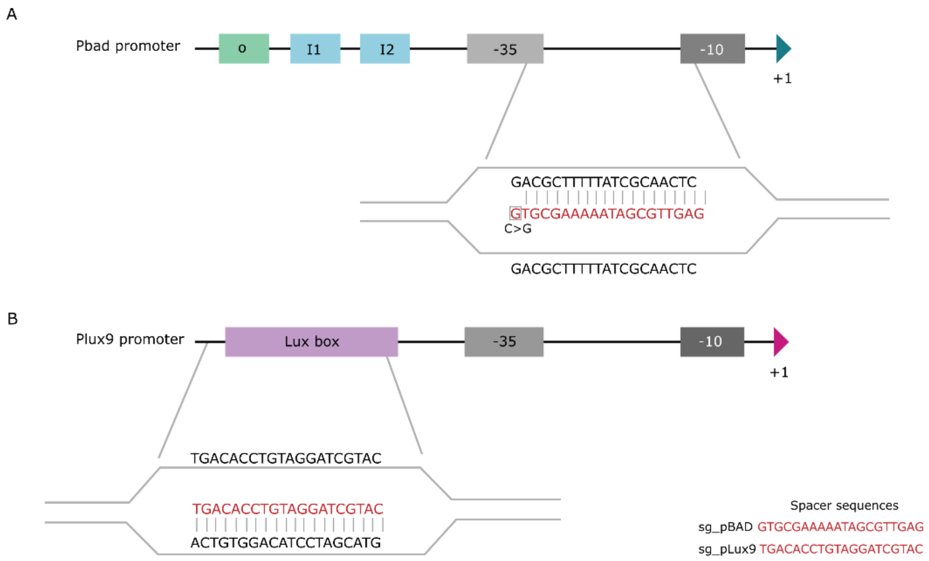

Figure S1. Design of guide RNAs targeting pBAD and pLux9 promoters. (**a**) Schematic of the pBAD promoter. Operator sites O, I1, and I2 are critical for AraC-mediated regulation. The -35 and -10 regions are shown as gray boxes, and the transcription start site (TSS) is marked by a green triangle. The sgRNA spacer (red) targets the region between the -35 and -10 boxes and includes a C>G mutation at the 5′ end adjacent to the PAM sequence. (**b**) Schematic of the pLux9 promoter. The -35 and -10 regions are also shown in gray, with the TSS indicated by a pink triangle. The sgRNA spacer (red) is designed to target a region overlapping the LuxR binding site (Lux box).

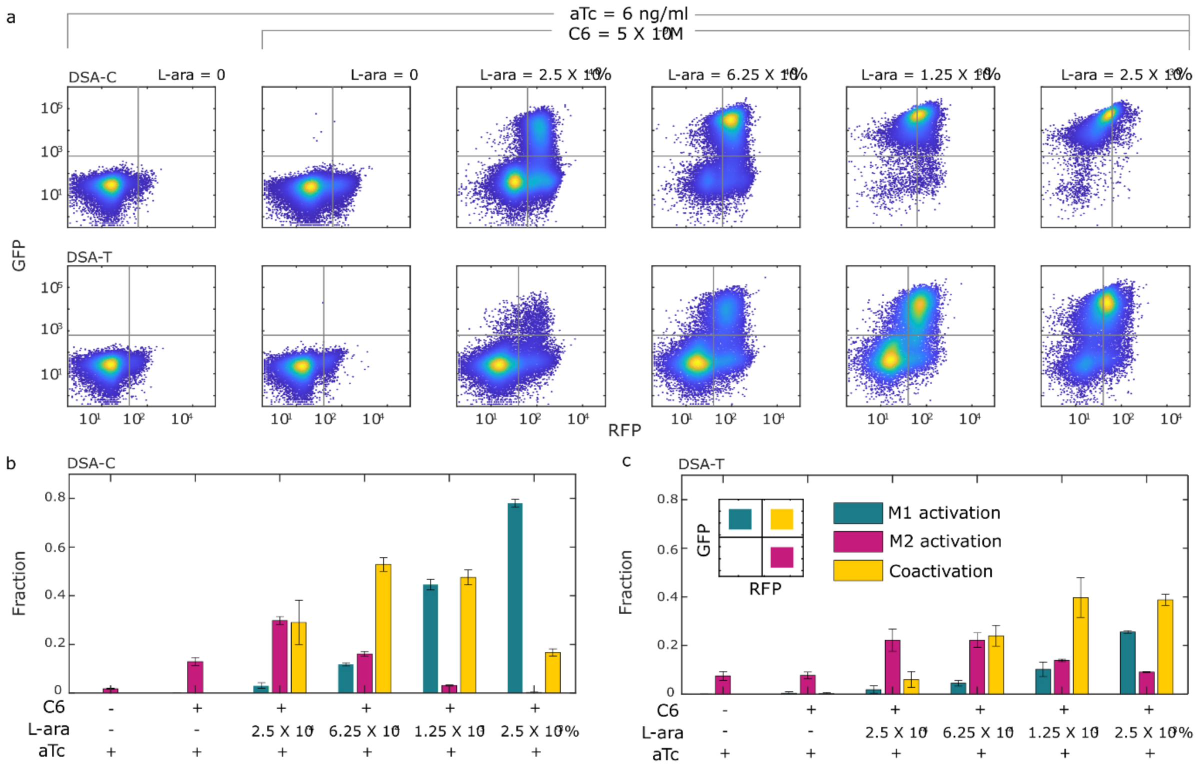

Figure S2. Mitigation of WTA resource competition in DSA circuits at fixed C6 dose and increasing L-ara. (a) Flow cytometry data illustrate cell state transitions in DSA-C (top) and DSA-T (bottom) with increasing L-ara concentrations, a fixed C6 (5 X 10^{-9} M), and constant aTc (6 ng/mL). For each sample, 10,000 events were recorded. The data shown represent one of three independent biological replicates. (b-c) The fraction of cells in M1 activation (green), M2 activation (pink), and coactivation (yellow) states in DSA-C (c) and DSA-T (d) across increasing L-ara concentrations. Data are presented as mean ± s.d., n = 3.

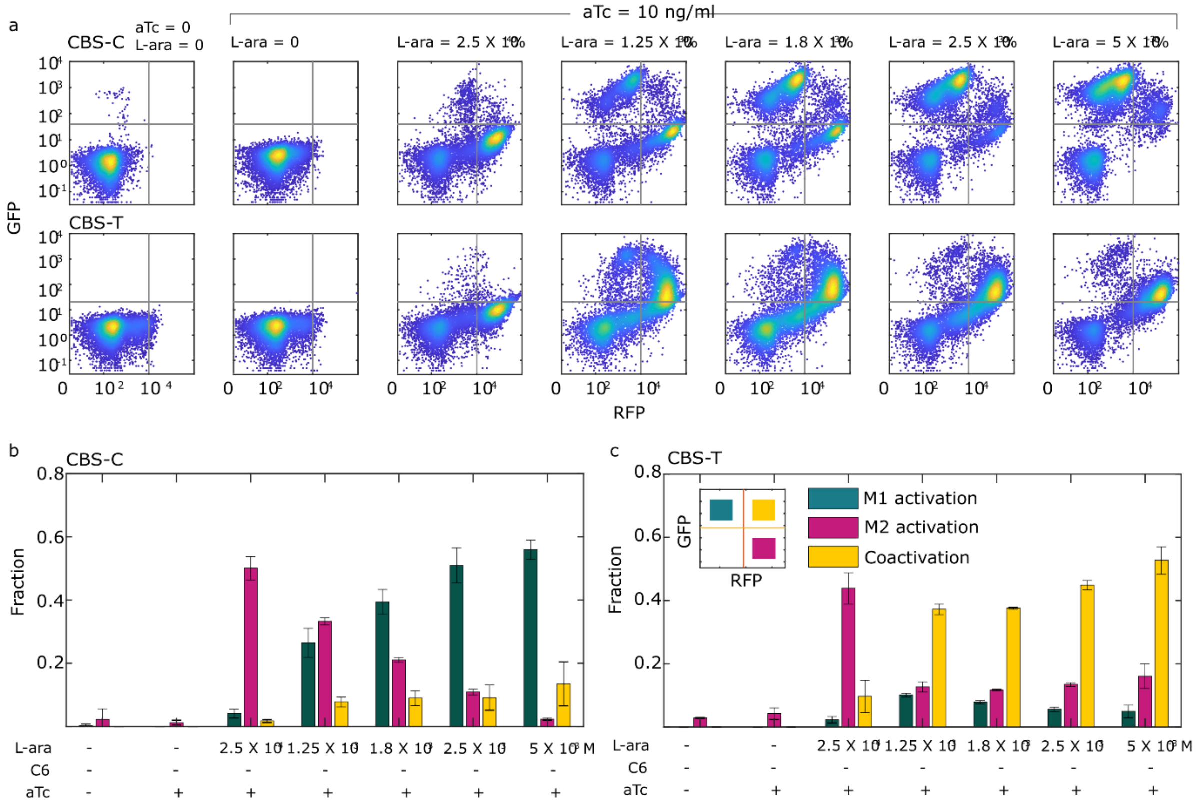

Figure S3. CRISPR-driven WTA mitigation in CBS circuits at a different level of dCas9. (a) Flow cytometry data illustrate cell state transitions in CBS-C (top) and CBS-T (bottom) with increasing L-ara concentrations and constant aTc (10 ng/mL). For each sample, 10,000 events were recorded. The data shown represent one of three independent biological replicates. (b-c) The fraction of cells in M1 activation (green), M2 activation (pink), and coactivation (yellow) states in CBS-C (d) and CBS-T (e) across increasing Lara concentrations. Data are presented as mean ± s.d., n = 3.

Supplemental Table 1. List of BioBrick parts and their sequences used in the study

BioBrick codes *(mutations if any)*	Part description	Sequence used in the study (5’→3’)
K206000 *(deletions of bases after the TSS)*	Strong pBad promoter	acattgattatttgcacggcgtcacactttgctatgccatagcaagatagtccataagattagcggatcctacctgacgctttttatcgcaactctctactgtttctccataccgtttttttgggctagc
R0062 *(A→T at -10 and G→T -11 relative to the TSS)*	A variant of pLux promoter – pLux9	acctgtaggatcgtacagggttacgcaagaaaatggtttgttatagtcgaataaa
P0440	pTet promoter	tccctatcagtgatagagattgacatccctatcagtgatagagatactgagcac
J23117	J23117 constitutive promoter	ttgacagctagctcagtcctagggattgtgctagc
J04031	Green Fluorescent Protein (GFP) with Lva tag	atgcgtaaaggagaagaacttttcactggagttgtcccaattcttgttgaattagatggtgatgttaatgggcacaaattttctgtcagtggagagggtgaaggtgatgcaacatacggaaaacttacccttaaatttatttgcactactggaaaactacctgttccatggccaacacttgtcactactttcggttatggtgttcaatgctttgcgagatacccagatcatatgaaacagcatgactttttcaagagtgccatgcccgaaggttatgtacaggaaagaactatatttttcaaagatgacgggaactacaagacacgtgctgaagtcaagtttgaaggtgatacccttgttaatagaatcgagttaaaaggtattgattttaaagaagatggaaacattcttggacacaaattggaatacaactataactcacacaatgtatacatcatggcagacaaacaaaagaatggaatcaaagttaacttcaaaattagacacaacattgaagatggaagcgttcaactagcagaccattatcaacaaaatactccaattggcgatggccctgtccttttaccagacaaccattacctgtccacacaatctgccctttcgaaagatcccaacgaaaagagagaccacatggtccttcttgagtttgtaacagctgctgggattacacatggcatggatgaactatacaaaaggcctgctgcaaacgacgaaaactacgctttagtagcttaa
E1010	Red Fluorescent Protein (RFP)	atggcttcctccgaagacgttatcaaagagttcatgcgtttcaaagttcgtatggaaggttccgttaacggtcacgagttcgaaatcgaaggtgaaggtgaaggtcgtccgtacgaaggtacccagaccgctaaactgaaagttaccaaaggtggtccgctgccgttcgcttgggacatcctgtccccgcagttccagtacggttccaaagcttacgttaaacacccggctgacatcccggactacctgaaactgtccttccccggaaggtttcaaatgggaacgtgttatgaacttcgaagacggtggtgttgttaccgttacccaggactcctccctgcaagacggtgagttcatctacaaagttaaactgcgtggtaccaacttcccgtccgacggtccggttatgcagaaaaaaaccatgggttgggaagcttccaccgaacgtatgtacccggaagacggtgctctgaaaggtgaaatcaaaatgcgtctgaaactgaaagacggtggtcactacgacgctgaagttaaaaccacctacatggctaaaaaaccggttcagctgccgggtgcttacaaaaccgacatcaaactggacatcacctcccacaacgaagactacaccatcgttgaacagtacgaacgtgctgaaggtcgtcactccaccggtgcttaa

C0040	TetR	atgtccagattagataaaagtaaagtgattaacagcgcattag<br>agctgcttaatgaggtcggaatcgaaggtttaacaacccgtaa<br>actcgcccagaagctaggtgtagagcagcctacattgtattggc<br>atgtaaaaaataagcgggctttgctcgacgccttagccattgag<br>atgttagataggcaccatactcacttttgccctttagaaggggaa<br>agctggcaagattttttacgtaataacgctaaaagttttagatgtg<br>ctttactaagtcatcgcgatggagcaaaagtacatttaggtaca<br>cggcctacagaaaaacagtatgaaactctcgaaaatcaatta<br>gcccttttatgccaacaaggtttttcactagagaatgcattatatgc<br>actcagcgctgtggggcattttactttaggttgcgtattggaagat<br>caagagcatcaagtcgctaaagaagaaagggaaacaccta<br>ctactgatagtatgccgccattattacgacaagctatcgaattatt<br>tgatcaccaaggtgcagagccagccttcttattcggccttgaatt<br>gatcatatgcggattagaaaaacaacttaaatgtgaaagtggg<br>tccgctgcaaacgacgaaaactacgctttagtagcttaataaca<br>ctgatagtgctagtgtagatcactaa
B0015	Double terminator	ccaggcatcaaataaaacgaaaggctcagtcgaaagactgg<br>gcctttcgttttatctgttgtttgtcggtgaacgctctctactagagtc<br>acactggctcaccttcgggtgggcctttctgcgtttata
B0034	Strong Ribosome Binding Site (RBS)	aaagaggagaaa
C0080<br>*(lva tag removed)*	AraC	atggctgaagcgcaaaatgatcccctgctgccgggatactcgtt<br>taacgcccatctggtggcgggtttaacgccgattgaggccaatg<br>gttatctcgatttttttatcgaccgaccgctgggaatgaaaggttat<br>attctcaatctcaccattcgcggtcagggggtggtgaaaaatca<br>gggacgagaatttgtctgccgaccgggtgatattttgctgttccc<br>gccaggagagattcatcactacggtcgtcatccggaggctcgc<br>gaatggtatcaccagtgggtttactttcgtccgcgcgcctactgg<br>catgaatggcttaactggccgtcaatatttgccaatacgggtttct<br>ttcgcccggatgaagcgcaccagccgcatttcagcgacctgttt<br>gggcaaatcattaacgccgggcaaggggaagggcgctattc<br>ggagctgctggcgataaatctgcttgagcaattgttactgcggc<br>gcatggaagcgattaacgagtcgctccatccaccgatggataa<br>tcgggtacgcgaggcttgtcagtacatcagcgatcacctggca<br>gacagcaattttgatatcgccagcgtcgcacagcatgtttgcctg<br>tcgccgtcgcgtctgtcacatcttttccgccagcagttagggatta<br>gcgtcttaagctggcgcgaggaccaacgcatcagccaggcg<br>aagctgcttttgagcactacccggatgcctatcgccaccgtcgg<br>tcgcaatgttggttttgacgatcaactctatttctcgcgagtatttaa<br>aaaatgcaccggggccagcccgagcgagttccgtgccggttg<br>tgaagaaaaagtgaatgatgtagccgtcaagttgtcataa
C0061	LuxI	atgactataatgataaaaaaatcggatttttggcaattccatcgg<br>aggagtataaaggtattctaagtcttcgttatcaagtgtttaagca<br>aagacttgagtgggacttagttgtagaaaataaccttgaatcag<br>atgagtatgataactcaaatgcagaatatatttatgcttgtgatga<br>tactgaaaatgtaagtggatgctggcgtttattacctacaacagg<br>tgattatatgctgaaaagtgtttttcctgaattgcttggtcaacaga<br>gtgctcccaaagatcctaatatagtcgaattaagtcgttttgctgt<br>aggtaaaaatagctcaaagataaataactctgctagtgaaatt<br>acaatgaaactatttgaagctatatataaacacgctgttagtcaa

		ggtattacagaatatgtaacagtaacatcaacagcaatagagc<br>gatttttaaagcgtattaaagttccttgtcatcgtattggagacaaa<br>gaaattcatgtattaggtgatactaaatcggttgtattgtctatgcct<br>attaatgaacagtttaaaaaagcagtcttaaatgctgcaaacga<br>cgaaaactacgctttagtagcttaa
C0062<br>*(S116A and M135I mutations were added)*	A variant of LuxR – LuxRG2C	atgaaaaacataaatgccgacgacacatacagaataattaat<br>aaaattaaagcttgtagaagcaataatgatattaatcaatgctta<br>tctgatatgactaaaatggtacattgtgaatattatttactcgcgat<br>catttatcctcattctatggttaaatctgatatttcaatcctagataat<br>taccctaaaaaatggaggcaatattatgatgacgctaatttaata<br>aaatatgatcctatagtagattattctaactccaatcattcaccaa<br>ttaattggaatatatttgaaaacaatgctgtaaataaaaaatctc<br>caaatgtaattaaagaagcgaaaacagcaggtcttatcactgg<br>gtttagtttccctattcatacggctaacaatggcttcggaatactta<br>gttttgcacattcagaaaaagacaactatatagatagtttattttta<br>catgcgtgtatgaacataccattaattgttccttctctagttgataat<br>tatcgaaaaataaatatagcaaataataaatcaaacaacgatt<br>taaccaaaagagaaaaagaatgtttagcgtgggcatgcgaa<br>ggaaaaagctcttgggatatttcaaaaatattaggttgcagtga<br>gcgtactgtcactttccatttaaccaatgcgcaaatgaaactcaa<br>tacaacaaaccgctgccaaagtatttctaaagcaattttaacag<br>gagcaattgattgcccatactttaaaaattaa

Supplemental Table 2. Gene circuits used in the study

Name	Gene construct
DSAC-T	K206000 + B0034 + GFP-lva + B0015 + K206000 + B0034 + araC + B0015 + K206000 + sg-pBAD + B0015 + Plux9 + B0034 + RFP-lva + B0015 + Plux9 + B0034 + luxRG2C + B0015 + Plux9 + sg-pLux9 + B0015 + J23117 + B0034 + TetR + B0015
DSAC-C	K206000 + B0034 + GFP-lva + B0015 + K206000 + B0034 + araC + B0015 + K206000 + NT + B0015 + Plux9 + B0034 + RFP-lva + B0015 + Plux9 + B0034 + luxRG2C + B0015 + Plux9 + NT + B0015 + J23117 + B0034 + TetR + B0015
CBS-T	K206000 + B0034 + GFP-lva + B0015 + K206000 + B0034 + araC + B0015 + K206000 + sg-pBAD + B0015 + Plux9 + B0034 + RFP-lva + B0015 + Plux9 + B0034 + luxRG2C + B0015 + Plux9 + sg-pLux9 + B0015 + J23117 + B0034 + TetR + B0015 + K206000 + B0034 + C0061 + B0015 + Plux9 + B0034 + araC + B0015
CBS-C	K206000 + B0034 + GFP-lva + B0015 + K206000 + B0034 + araC + B0015 + K206000 + NT + B0015 + Plux9 + B0034 + RFP-lva + B0015 + Plux9 + B0034 + luxRG2C + B0015 + Plux9 + NT + B0015 + J23117 + B0034 + TetR + B0015 + K206000 + B0034 + C0061 + B0015 + Plux9 + B0034 + araC + B0015
NCR cassette	P0440 + dCas9 + B0015

Supplemental Table 3. Oligonucleotides used in this study

Sequence (5’→3’)	Oligonucleotide description
ataagagttgcgataaaaagcgtg	Forward gRNA targeting pBAD
aaccacgctttttatcgcaactct	Reverse gRNA targeting pBAD
aaatgacacctgtaggatcgtac	Forward gRNA targeting pLux9
aacgtacgatcctacaggtgtca	Reverse gRNA targeting pLux9
tctggaattcgcggccgcttctagagtgacacctgtaggatcgtacaggg	Forward addition of TGAC to plux9 (EcoRI and XbaI)
ggactgcagcggccgctactagtacgctcttcttatggagaaacagtagagag	Reverse deletion of pBAD bases after TSS site (SapI, SpeI, and PstI)
ctagagtgacacctgtaggatcgtacagggttacgcaagaaaatggtttgttatagtcgaataaaagaagagcacaggctcttct	Forward Plux9 and non-targeting guide (SapI and SapI)
aacagaagagcctgtgctcttcttttattcgactataacaaaccattttcttgcgtaaccctgtacgatcctacaggtgtcact	Reverse Plux9 and non-targeting guide (SapI and SapI)
atgctgatttgtttttggcagc	Forward dCas9 specific primer
actccttggagaatccgcct	Reverse dCas9 specific primer
tctggaattcgcggccgcttctagagaaagaggagaaaggatctat	Forward addition of BioBrick restriction sites to dCas9 (EcoRI and XbaI)
ggactgcagcggccgctactagtacgcagaaaggcccacccgaa	Reverse addition of BioBrick restriction sites to dCas9 (SpeI and PstI)
ggactgcagcggccgctactagtattattaagctactaaagcgtagttttcgtcgtttgcagcagcaccggtggagtgacgac	Addition of lva tag to RFP (SpeI and PstI)
tctagaacaggctcttctgttttagagctagaaatagc	Forward dCas9 gRNA scaffold (XbaI and SapI)
ggactgcagcggccgctactagtagcaccgactcggtgccactt	Reverse addition of BioBrick restriction sites to dCas9 gRNA scaffold (SpeI and PstI)
